# Development of graded prognostic assessment for breast Cancer brain metastasis incorporating extracranial metastatic features: a retrospective analysis of 284 patients

**DOI:** 10.1186/s12885-024-12983-3

**Published:** 2024-10-10

**Authors:** Yan Wang, Hangcheng Xu, Qiang Sa, Li Li, Yiqun Han, Yun Wu, Yiran Zhou, Binghe Xu, Jiayu Wang

**Affiliations:** 1 Department of Medical Oncology, National Clinical Research Center for Cancer/Cancer Hospital, National Cancer Center, Chinese Academy of Medical Sciences and Peking Union Medical College, No. 17, Panjiayuan Nanli, Chaoyang District, Beijing, 100021 China; 2 Department of Medical Records, National Clinical Research Center for Cancer/Cancer Hospital, National Cancer Center, Chinese Academy of Medical Sciences and Peking Union Medical College, No. 17, Panjiayuan Nanli, Chaoyang District, Beijing, 100021 China

**Keywords:** Breast cancer, Brain metastasis, Extracranial metastasis, GPA model, Prognosis

## Abstract

**Background:**

Breast cancer brain metastasis (BCBM) is associated with poor survival outcomes and reduced quality of life. The Graded Prognostic Assessment (GPA) score model serves as a well-established tool for predicting the prognosis of BCBM. Notably, the presence of extracranial metastasis (ECM) is considered as a significant prognostic factor in the breast GPA model. This study aims to further refine other features of ECM to enhance the prognostic prediction for BCBM.

**Methods:**

This study included all inpatients diagnosed with BCBM at the Cancer Hospital, Chinese Academy of Medical Sciences, from January 2010 to July 2021. Baseline characteristics of patients were compared based on features of ECM, including the presence, number, location, and control status of metastases. Overall survival (OS) were compared using the Kaplan–Meier method with log-rank tests. Cox regression analyses were conducted to identify significant prognostic factors. The aforementioned ECM features were incorporated into the original Breast-GPA model to enhance its prognostic accuracy. The concordance index (C-index) and restricted mean survival time (RMST) were utilized to evaluate and compare the predictive accuracy of the updated and original survival models.

**Results:**

284 patients with BCBM were included in the study. Kaplan–Meier survival curves suggested that patients without ECM when diagnosed with BCBM showed better survival (*p* = 0.007). In the subgroups with ECM, more than 3 organs involved, both bone and visceral metastasis and progressive ECM portended dismal OS (*p* = 0.003, 0.001 and <0.001). Multivariate analysis demonstrated that molecular subtype, presence of ECM, and number of brain metastasis significantly influenced OS after BCBM. By modifying the current GPA model to include more precise characteristics of ECM, the predictive accuracy was further enhanced as indicated by the C-index and RMST curve.

**Conclusions:**

More ECM sites, both bone and visceral invasion and uncontrolled ECM were dismal prognostic factors for survival outcomes of BCBM patients. A new Breast-GPA model with better predictive effect was constructed.

**Supplementary Information:**

The online version contains supplementary material available at 10.1186/s12885-024-12983-3.

## Introduction

Breast cancer is the second frequent malignant tumors of suffering from brain metastasis, although the incidence was always thought to be underestimated [1]. Upon diagnosis, breast cancer brain metastasis (BCBM) portends poor survival outcomes and the blood brain barrier also poses a significant clinical quandary, given limited penetration of many chemotherapies and lack of information regarding their central nervous system efficacy [2, 3]. With further research, more and more therapeutic methods had been developed and utilized in the clinical practice, including surgery, stereotactic radiosurgery (SRS), whole-brain radiation therapy (WBRT), chemotherapy, targeted therapy and immunotherapy [4]. The advanced treatment strategies and prolonged life expectancy emphasized the significance of predicting prognosis to optimize physicians’ choice, patients’ decision and palliative treatment.

Apart from the features of brain lesions, extracranial metastasis (ECM) was also related to the occurrence risk and long-term survival for BCBM. The National Comprehensive Cancer Network guideline recommended brain magnetic resonance imaging scan for the patients with recurrent or metastatic breast cancer if suspicious symptoms of central nervous system occur [5]. A study based on the Surveillance, Epidemiology, and End Results database concluded that when using bone metastasis as a reference, lung metastasis increased the risk of brain metastasis in patients with triple negative breast cancer [6]. Other retrospective study also demonstrated the predictive value of ECM pattern, which was included in their newly established risk model for BCBM [7].

As for the predictive value of ECM for overall survival (OS) after BCBM, numerous studies have explored [8–13] and the most authoritative was the Graded Prognostic Assessment (GPA) tool. The Breast-GPA tool only included the age of diagnosis, Karnofsky performance score (KPS) and molecular subtype for survival predication in 2012 [14]. With gradual update, the modified GPA incorporated the number of brain lesions in 2015 [15], and the 2020 updated version included the presence of ECM [16]. Regarding ECM, there are many other characteristics, such as the number of ECM organs, the specific location of ECM and whether extracranial diseases are controlled or progressive. All these features were also confirmed to be closely related with the long-term mortality of BCBM patients in other analysis [13, 17–19].

In light of the aforementioned factors, we set up the database of BCBM patients treated in our institution, intending to clarify the impact of the presence, quantity, site and control status of ECM on the prognostic outcomes to optimize the GPA model.

## Methods

### Enrolled cohort

We conducted a retrospective cohort study enrolling patients diagnosed with BCBM at the Cancer Hospital, Chinese Academy of Medical Sciences (CHCAMS) from January 1st, 2010, to July 1st, 2021. BCBM was diagnosed either at the initial diagnosis of breast cancer or during follow-up after treatment. The study was approved by the Ethics Committee of CHCAMS (the ethical approval number: NCC4835) and adhered to the Helsinki Declaration. We excluded cases with duplicate registration, inadequate neuroimaging or pathological evidence of brain metastasis, and patients with other primary malignancies or contralateral breast cancer. All BCBM patients who did not meet these exclusion criteria were included in the study. The last follow-up was on July 1st, 2023.

### Data collection

We documented detailed clinicopathological data for all participants, including age at first diagnosis, menopausal status, KPS or Eastern Cooperative Oncology Group (ECOG) performance status, pathological grade, TNM stage, hormone receptor (HR) status, and human epidermal growth factor receptor 2 (HER2) status. Treatment history for primary breast cancer, including neoadjuvant therapy, surgical interventions, and adjuvant chemotherapy or radiotherapy, were recorded. Disease-free survival was calculated as the duration from the date of radical surgery to the occurrence of any disease progression, including local recurrence, distant metastasis, the development of new tumors, or death due to the tumor.

For BCBM, we extracted data encompassing ECM characteristics, diagnostic mode (symptomatic diagnosis or occasional findings in imaging), brain lesion features (including number, location and maximum diameter), and administered therapy (including local and systemic therapy). In detailing ECM, we noted the presence (present or absent), number of involved organs (0, 1–3, or ≥ 4), location (bone-only, visceral-only, or both bone and visceral), and control status (controlled or uncontrolled) of the metastases. These ECM features were then analyzed across different subgroups. OS after brain metastasis was determined from the time of BCBM diagnosis to either the occurrence of breast cancer-related death or the date of the last follow-up, whichever occurred first.

### Statistical analysis

The baseline characteristics across different subgroups were compared using the chi-square test or Fisher’s exact test for categorical variables and the t-test or Mann-Whitney U test for continuous variables, as appropriate. Survival outcomes were analyzed using the Kaplan-Meier method, and differences between groups were assessed using the log-rank test. A Cox proportional hazards model was employed for multivariate analysis, including all clinicopathological features with a univariate *p*-value < 0.1. To compare the prognostic performance of the original and updated Breast-GPA models, both the restricted mean survival time (RMST) at a fixed time point of 24 months and the concordance index (C-index) were employed. The RMST is a measure of the average survival time of different score groups within a specified time period. A two-tailed *p*-value < 0.05 was considered statistically significant. All analyses were performed using SPSS version 26.0 (SPSS Inc, Chicago, IL, USA) and the “ggplot”, “survminer”, “survival”, “survRM2” packages of R software (version 4.2.3; http://www.r-project.org).

## Results

### Baseline characteristics

Totally, 284 patients diagnosed with BCBM were included in this study. In analyzing baseline characteristics of BCBM patients with or without ECM, we observed that those diagnosed with one or more extracranial lesions prior to brain metastasis had a higher incidence of M1 stage at initial breast cancer diagnosis (16% vs. 4%, *p* = 0.040), underwent more radical mastectomies for their primary tumors (71% vs. 58%, *p* = 0.044), and were more frequently detected through occasional imaging screenings (58% vs. 29%, *p*<0.001) (Supplementary Table 1). Additionally, patients with ECM tended to have a higher number of brain lesions (69% vs. 46%, *p* = 0.003), which reduced the likelihood of undergoing neurosurgical interventions (11% vs. 27%, *p* = 0.003).

Further analysis of the specific number of extracerebral organs showed similar baseline comparisons (Supplementary Table 2). Notably, patients with involvement of more than 4 organs were less likely to have HER2-positive disease (*p* = 0.044) and thus rarely received anti-HER2 targeted therapy (*p* = 0.024). Regarding the baseline comparison between patients with controlled versus uncontrolled extracranial disease, differences were observed in the surgical intervention of primary tumors and brain metastases (*p* = 0.041 and <0.001, respectively), disease-free survival (*p* = 0.001), and the diagnostic mode for BCBM (*p*<0.001) (Supplementary Table 3).

### Survival outcomes

**Fig. 1:**
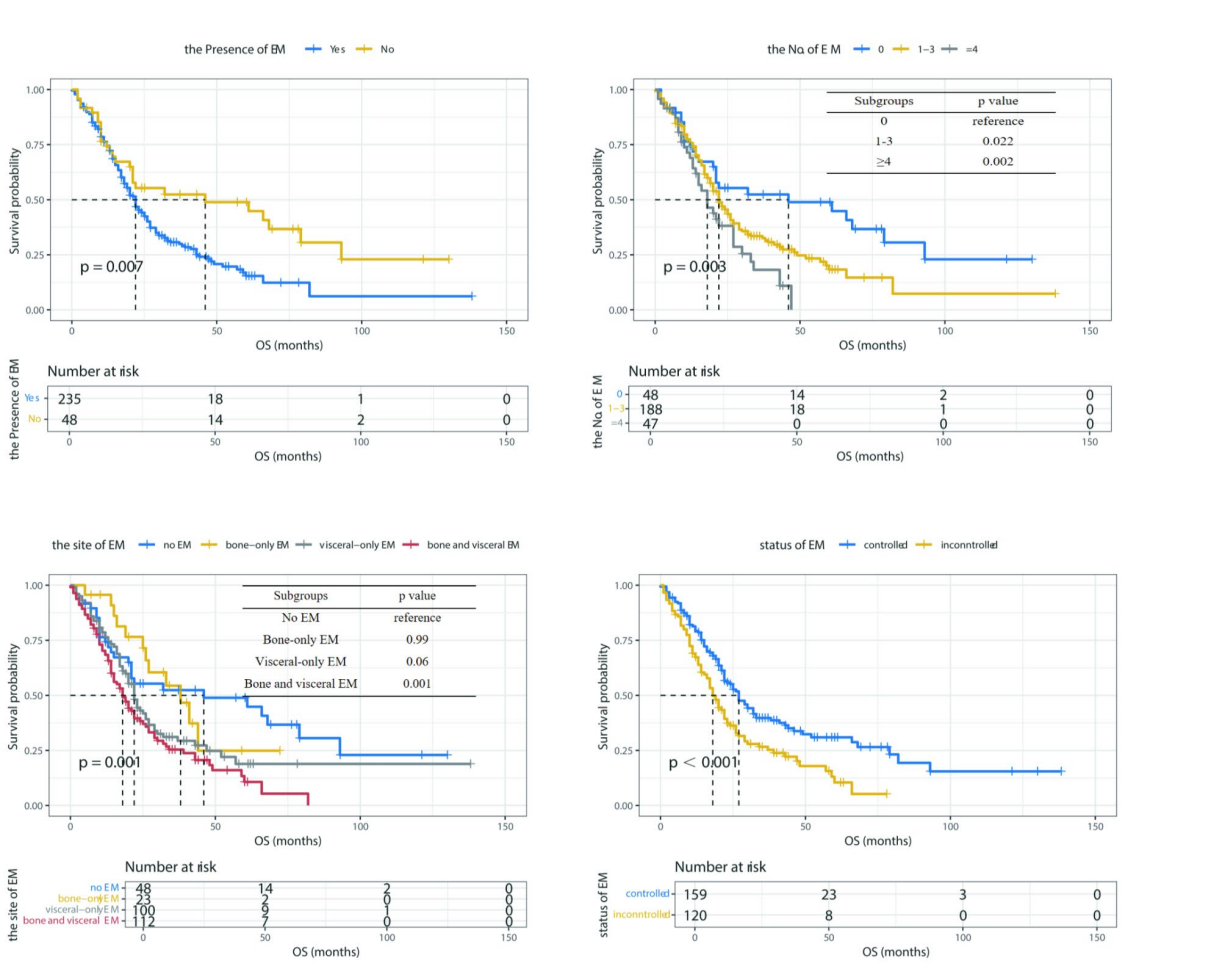
Kaplan-Meier curves for OS of BCBM patients based on ECM characteristics Note: The subfigures were categorized by (**A**) the presence of ECM, (**B**) the number of involved ECM organs, (**C**) the location of ECM, and (**D**) the control status of ECM **Abbreviations:** ECM: extracranial metastasis; OS: overall survival; BCBM: breast cancer brain metastasis

The study compared the long-term survival of BCBM patients across subgroups, differentiated by factors like the presence, number, location and control status of ECM. Patients without ECM showed a significantly longer median OS of 46 months, compared to 22 months in those with ECM (*p* = 0.007, Fig. 1A). Furthermore, an increase in the number of affected extracranial organs correlated with poorer prognosis. Specifically, patients with more than four affected extracranial organs had a median OS of 18 months, considerably lower than 22 months for those with 1–3 metastatic sites and 46 months for patients without ECM (*p* = 0.003, Fig. 1B). Analyzing the ECM locations, significant differences in OS were observed between patients with bone-only, visceral-only, and both bone and visceral metastases (*p* = 0.001, Fig. 1C). As for the control status of ECM, patients with controlled ECM showed a clear trend in favorable OS compared to those with uncontrolled ECM (*p*<0.001, Fig. 1D).

To further elucidate the prognostic factors influencing OS of BCBM patients, we performed a Cox regression analysis (Table 1). We included all clinicopathological features with univariate *p*-value lower than 0.1 in the multivariate study. These features included the age at breast cancer diagnosis, HR status, HER2 status, the presence, number, and status of ECM, the number and size of BM, surgical intervention for BM and endocrine and targeted therapy post-BCBM. Notably, the HR and HER2 status, presence of ECM, number of BM and targeted therapy after BM were identified as independent prognostic factors for BCBM in the multivariate analysis, with respective *p*-values of 0.009, 0.002, 0.038,0.001 and 0.012.

**Table 1:** Univariate and multivariate analysis of variables correlated with OS after brain metastasis

Variables	Univariate Analysis		Multivariate Analysis	
	Hazard Ratio (95% CI)	*p*-value	Hazard Ratio (95% CI)	*p*-value
**Age**,** years**		**0.066**		0.116
<40	Reference		Reference	
40–59	1.33 (0.95–1.86)	0.099	1.20 (0.80–1.79)	0.394
60–79	1.79 (1.01–2.98)	0.025	1.86 (1.03–3.37)	0.041
**Menopausal Status**		0.280		
Premenopausal	Reference			
Postmenopausal	1.17 (0.88–1.56)	0.280		
**Performance Status**		0.175		
KPS 80–100 / ECOG 0–1	Reference			
KPS ≤ 70 / ECOG 2–3	1.30 (0.90–1.89)	0.164		
Unknown	0.86 (0.60–1.23)	0.393		
**Grade**		0.138		
Grade I-II	Reference			
Grade III	1.43 (0.96–2.01)	0.053		
Unknown	1.08 (0.78–1.50)	0.670		
**T Stage**		0.542		
Tis/T0-2	Reference			
T3-T4	0.78 (0.49–1.23)	0.281		
Unknown	0.92 (0.66–1.28)	0.627		
**N Stage**		0.762		
N0-N1	Reference			
N2-N3	0.94 (0.69–1.30)	0.722		
Unknown	0.86 (0.58–1.28)	0.466		
**M Stage**		0.815		
M0	Reference			
M1	1.05 (0.70–1.58)	0.815		
**TNM Stage**		0.741		
Stage 0-II	Reference			
Stage III-IV	0.94 (0.68–1.28)	0.676		
Unknown	0.85 (0.57–1.28)	0.443		
**HR Status**		**0.058**		**0.009**
Positive	Reference		Reference	
Negative	1.33 (0.99–1.78)	0.058	1.86 (1.16–2.97)	0.009
**HER2 Status**		**0.035**		**0.002**
Positive	Reference		Reference	
Negative	1.37 (1.02–1.84)	0.035	1.77 (1.22–2.57)	0.002
**Neoadjuvant Therapy**		0.259		
No	Reference			
Yes	0.81 (0.57–1.16)	0.259		
**Surgery**		0.108		
No	Reference			
Yes-Radical Mastectomy	0.61 (0.37–1.01)	0.053		
Yes-Conserving Surgery	0.56 (0.32–0.98)	0.042		
**Adjuvant Chemotherapy**		0.126		
No	Reference			
Yes	0.98 (0.63–1.52)	0.932		
Without Surgery	1.66 (0.89–3.11)	0.112		
**Adjuvant Radiotherapy**		0.116		
No	Reference			
Yes	0.94 (0.69–1.28)	0.695		
Without Surgery	1.59 (0.54–2.65)	0.075		
**Presence of ECM**		**0.008**		**0.038**
Absent	Reference		Reference	
Present	1.77 (1.16–2.71)	0.008	1.84 (1.03–3.27)	0.038
**Number of ECM**		**0.004**		0.110
Without ECM	Reference		Reference	
1–3 sites	1.67 (1.08–2.57)	0.020	1.83 (1.02–3.26)	0.042
≥4 sites	2.41 (1.43–4.07)	0.001	1.96 (0.99–3.90)	0.055
**Status of ECM**		**0.004**		0.088
Controlled	Reference		Reference	
Uncontrolled	1.67 (1.18–2.37)	0.004	1.36 (0.96–1.94)	0.088
**Diagnostic Mode**		0.739		
Symptomatic Diagnosis	Reference			
Occasional in Imaging	1.05 (0.79–1.40)	0.739		
**Number of BM**		**<0.001**		**0.001**
1	Reference		Reference	
≥2	1.97 (1.40–2.75)	<0.001	1.89 (1.28–2.79)	0.001
**Maximum of BM Diameter**		**0.052**		0.070
<3 cm	Reference		Reference	
≥3 cm	0.82 (0.54–1.25)	0.350	1.14 (0.70–1.86)	0.600
Unknown	1.32 (0.97–1.80)	0.079	1.53 (1.06–2.19)	0.022
**Radiotherapy for BM**		0.106		
No Radiotherapy	Reference			
SRS alone	0.79 (0.52–1.22)	0.286		
WBRT alone	1.26 (0.86–1.83)	0.238		
SRS plus WBRT	0.96 (0.56–1.66)	0.888		
**Surgical Intervention for BM**		**0.001**		0.065
No	Reference		Reference	
Yes	0.46 (0.28–0.74)	0.001	0.57 (0.31–1.04)	0.065
**Chemotherapy after BM**		0.475		
No	Reference			
Yes	0.87 (0.60–1.27)	0.475		
**Endocrine Therapy after BM**		**0.038**		0.051
ET for HR + tumors	Reference		Reference	
no ET for HR + tumors	1.46 (0.97–2.19)	0.071	1.57 (1.00-2.49)	0.051
HR- tumors	1.71 (1.13–2.59)	0.011	/	
**Targeted Therapy after BM**		**0.004**		**0.012**
TT for HER2 + tumors	Reference		Reference	
no TT for HER2 + tumors	2.00 (1.26–3.19)	0.003	2.00 (1.16–3.42)	0.012
HER2- tumors	1.57 (1.13–2.19)	0.008	/	

**Note:** P-values lower than 0.1 in the univariate analysis and 0.05 in the multivariate analysis were marked in bold. Due to an overlap in the definitions between the potential prognostic factors “presence of ECM” and “number of ECM”, they were not incorporated simultaneously in the multivariate analysis. Instead, the “number of ECM” was included in the final multivariate model in place of the “presence of ECM”

**Abbreviations:** CI: confidence interval; KPS: Karnofsky performance status; ECOG: Eastern Cooperative Oncology Group; HR: hormone receptor; HER2: human epidermal growth factor receptor 2; ECM: extracranial metastasis; BM: brain metastasis; SRS: stereotactic radiosurgery; WBRT: whole-brain radiotherapy; ET: endocrine therapy; TT: targeted therapy

### New Breast-GPA model construction

**Fig. 2:**
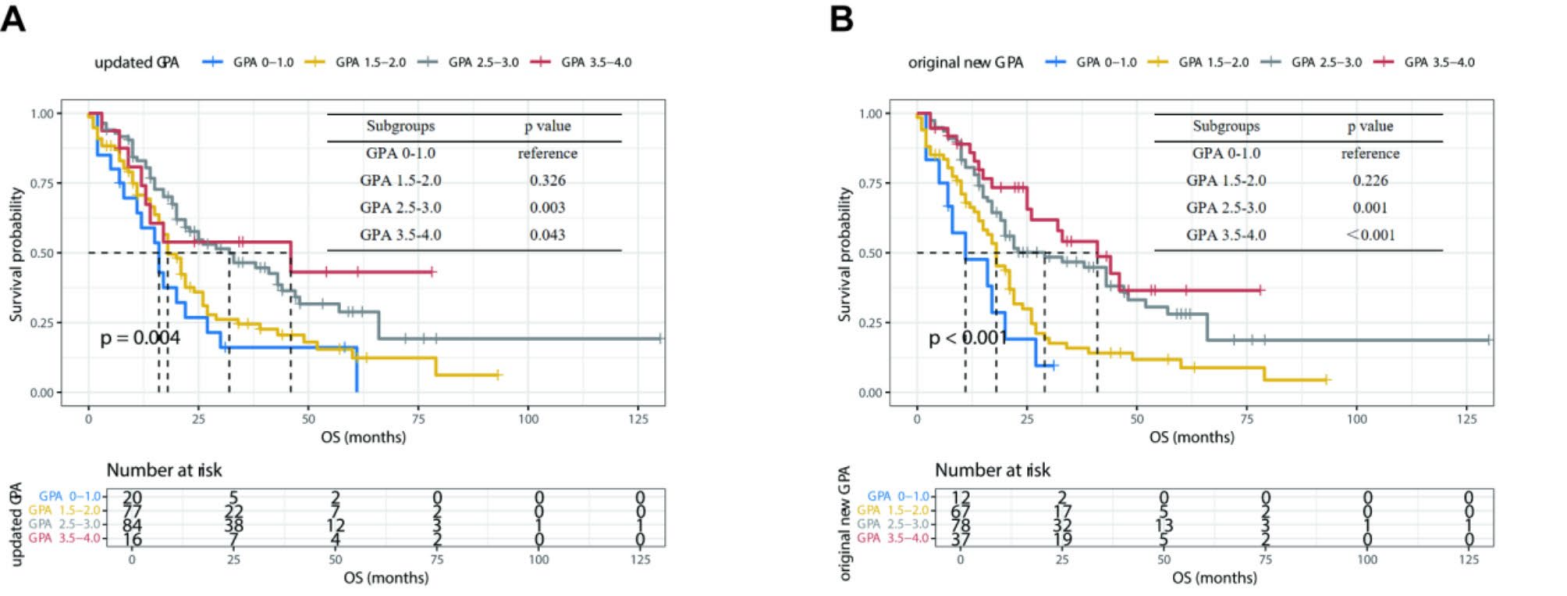
Kaplan-Meier curves for OS of BCBM patients by Breast-GPA models **Note:** The subfigures were categorized by (**A**) the original Breast-GPA model, and (**B**) the new Breast-GPA model. **Abbreviations:** GPA: the Graded Prognostic Assessment; OS: overall survival; BCBM: breast cancer brain metastasis.

**Fig. 3:**
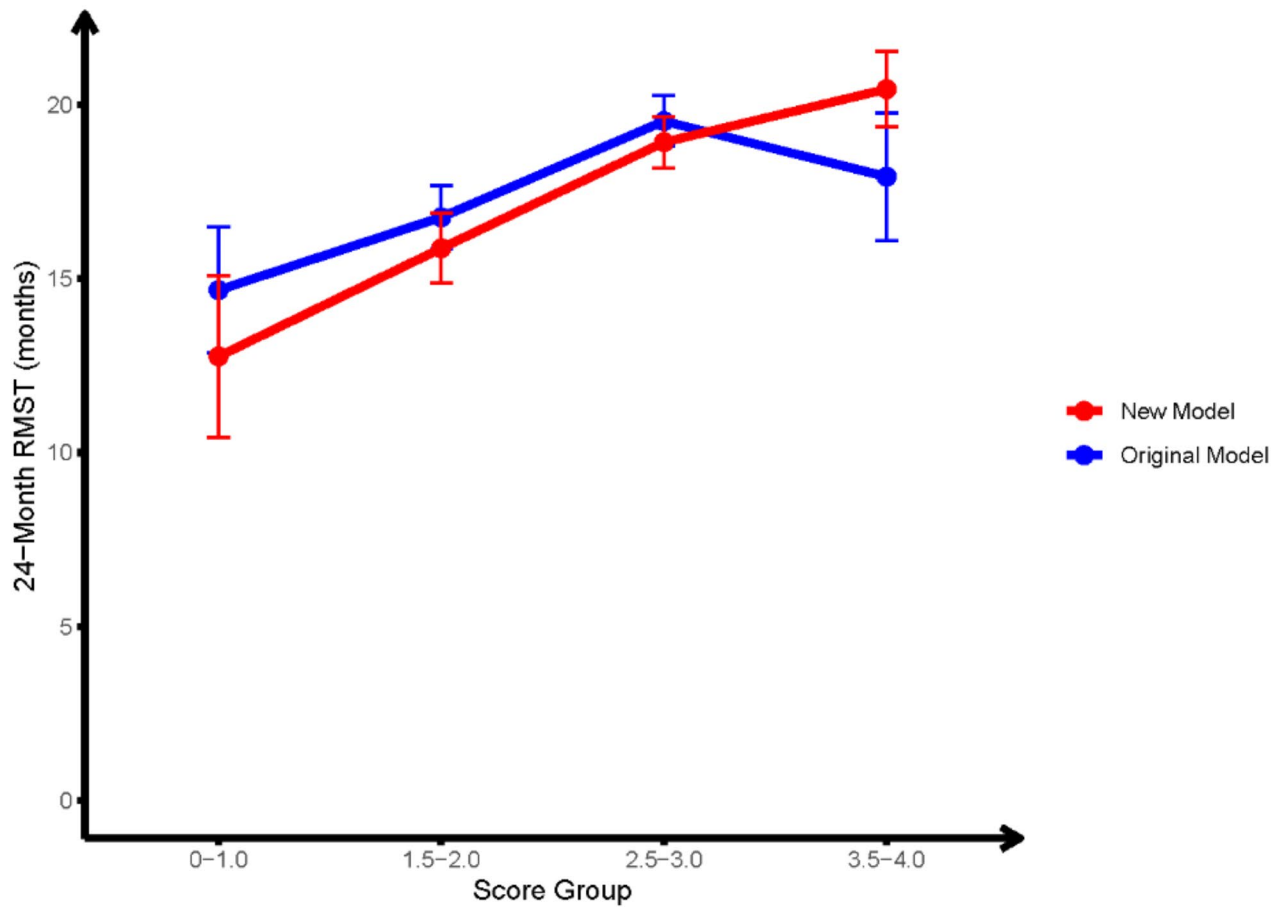
Comparison of 24-Month RMST between original and new breast-GPA Models **Note:** Plot of RMST curves for the original Breast-GPA model (blue), and the new Breast-GPA model (red). The error bars indicate the standard error associated with each RMST value Abbreviations: RMST: restricted mean survival time; GPA: Graded Prognostic Assessment; BCBM: breast cancer brain metastasis

According to the previous updated Breast-GPA scoring model (Table 2), all the enrolled patients were divided into four distinct subgroups. Significant differences were observed between different scoring subgroups (*p* = 0.004, Fig. 2A). It was evident that higher GPA scores were associated with more favorable OS in patients. Considering the number and control status of ECM were independent prognostic factors in the multivariate Cox analysis, we constructed a new Breast-GPA scoring model by incorporating these factors (Table 3). The new Breast-GPA model focused not only the presence, but also the quantity and control status of ECM. Cases with 3 or less controlled ECM were scored with 0.5, while those with more than 3 or progressive ECM were marked with 0. Survival outcomes were further measured by the new Breast-GPA model. In this model, the median OS of subgroups with a score of 3.5-4.0, 2.5-3.0, 1.5-2.0 and 0–1.0 were 41 months, 29 months, 18 months and 11 months, respectively (*p*<0.001, Fig. 2B). The C-index for the original Breast-GPA model was 0.582, whereas the C-index for the new Breast-GPA model was 0.616. The estimated 24-RMST for each score group in the two models were shown in the Supplementary Table 4. The RMST curve indicated that the new model had improved accuracy since the RMST values generally increased with higher Breast-GPA scores (Fig. 3).

**Table 2:** The definition of original Breast-GPA model

Factor	0	0.5	1	1.5	2
KPS	≤ 60	70–80	90–100	NA	NA
Age	≥ 60	<60	NA	NA	NA
Number of BM	≥ 2	1	NA	NA	NA
ECM	Present	Absent	NA	NA	NA
Subtype	Basal	Luminal A	NA	HER2 or Luminal B	NA

**Note:** The tumor subtypes were categorized into four distinct groups including basal-like subtype (triple-negative), luminal A subtype (ER/PR-positive and HER2-negative), HER2 subtype (ER/PR-negative and HER2-positive), and luminal B subtype (triple-positive)

**Abbreviations:** GPA: the Graded Prognostic Assessment; KPS: Karnofsky performance status; NA: not applicable; BM: brain metastasis; ECM: extracranial metastasis; ER: estrogen receptor; PR: pro gesterone receptor; HER2: human epidermal growth factor receptor 2

**Table 3:** The definition of new Breast-GPA model

Factor	0	0.5	1	1.5	2
KPS	≤ 60	70–80	90–100	NA	NA
Age	≥ 60	<60	NA	NA	NA
Number of BM	≥ 2	1	NA	NA	NA
ECM	>3 or progressive	≤ 3 and stable	NA	NA	NA
Subtype	Basal	Luminal A	NA	HER2 or Luminal B	NA

**Note:** The tumor subtypes were categorized into four distinct groups including basal-like subtype (triple-negative), luminal A subtype (ER/PR-positive and HER2-negative), HER2 subtype (ER/PR-negative and HER2-positive), and luminal B subtype (triple-positive)

**Abbreviations:** GPA: the Graded Prognostic Assessment; KPS: Karnofsky performance status; NA, not applicable; BM: brain metastasis; ECM: extracranial metastasis; ER: estrogen receptor; PR: progesterone receptor; HER2: human epidermal growth factor receptor 2

## Discussion

With the number of multi-disciplinary and patient-tailored therapeutic strategies increases exponentially, BCBM is not regarded as uncurable disease. Among a great number of treatment regimens, it is crucial to select the optimal therapeutic strategies in appropriate time. Therefore, there is an urgent requirement for prognostic models to guide clinical practice.

The gradually updated Breast-GPA prognosis model validated that the presence of ECM had impact on the prognosis of BCBM patients. According to this standard model, patients in our institution with higher GPA scores were confirmed with longer survival period. The Kaplan-Meier survival curves of different GPA score groups clustered into separate lines when classified into four groups (GPA 0–1.0, 1.5-2.0, 2.5-3.0 and 3.5-4.0), of which the median survival time were 16months, 18months, 32 months and 46 months respectively. It could be found that the life expectancy of patients in our constitution was comparatively longer than the GPA model [16], of which the OS after brain metastasis were 6months, 13 months, 24 months and 36 months in the corresponding GPA layers. This tendency could be explained by the improved therapeutic methods and sufficient supportive treatments for patients in the advanced stage, indicating that a new prognostic model is warranted in the current clinical practice.

Numerous previous studies have posited that many characteristics of ECM had impact on the prognosis of BCBM patients. Some researches revealed the large quantity of ECM organs as dismal prognosis factors [17–20]. Others explored the impact of exact ECM sites on long-term survival. The results suggested that the presence of lung [18, 20] or liver metastasis [20] exhibited a propensity for limited life expectany and were incorporated to establish prognostic models. Besides, the control status of extracranial lesions was also proven to be influential for the prognosis of BCBM patients [21, 22]. Considering the definition of quantity and location of extracranial metastases overlap each other, for example “both visceral and bone metastasis” portends larger number of ECM, these two aspects could not be included in the prognostic model simultaneously. We ultimately took the quantity and control status of ECM into consideration to renew the current Breast-GPA model [16], namely new Breast-GPA scoring model. The newly established GPA model no longer determined the survival outcomes of BCBM patients based on the presence or absence of extracranial metastases. Instead, patients with less than 3 stable ECM organs were marked with 0.5 points for GPA score, equivalent to those without ECM. This modification maintained the overall scoring structure of the Breast-GPA model. The discrepancy of survival curves in four GPA groups were much more conspicuous. Survival outcomes were further assessed using both the C-index and RMST at 24 months. The C-index for the updated Breast-GPA model was higher, and the differences in RMST across each scoring group were more obvious, as demonstrated by the RMST curve. These findings indicate that the updated model offers improved predictive accuracy compared to the original model. Depending on these results, clinical therapeutics for BCBM patients with limited number of controlled ECM were supposed to be relatively optimistic. For these patients, the clinical practice should focus more on the intracranial lesions, such as the craniocerebral operations, SRS and WBRT. In contrast, the population with multiple progressive visceral metastases were predicted with dismal prognosis, who require more aggressive systematic treatments and close surveillance after initial diagnosis of BCBM.

Except for the features of ECM, other characteristics were also verified to be independent prognostic factors for BCBM patients, including HR and HER2 status, the number of brain metastasis and targeted therapy after BCBM. The negative impact of HR and HER2 negativity on BCBM patients was validated in the Breast-GPA model [16], which was in concordance with other studies [23–25]. As for the quantity of brain metastasis, limited number of brain lesions was also associated with the feasibility of neurosurgical interventions and local radiotherapy, leading to prolonged OS. Moreover, patients with HER2-positive tumors who received targeted therapy after BCBM revealed much better prognosis than those without anti-HER2 treatment. These findings suggested that the HER2 targeted drugs were highly effective for BCBM patients. Of note, both tyrosine kinase inhibitor [26, 27] and antibody-drug conjugate [28, 29] were validated efficacious for BCBM patients despite the existence of blood brain barrier. TNM staging did not show significant prognostic value in the univariate or multivariate analyses. This may be due to the advanced disease stage of the patient cohort, where brain metastasis has a more dominant impact on prognosis than the primary tumor’s stage.

Undeniably, there exist several limitations. Firstly, this study is based on the population of one single institution and implemented retrospectively, which has some intrinsic deficiency. In details, the confounding, selection and information bias cannot be avoided utterly in the research process. Secondly, the progressive or stable status of extracranial disease was evaluated subjectively, restricted by the deficiency of official definition. Notably, three co-authors finished the assessment separately and discussed the inconsistent opinions. Plenty of other studies have also suggested the status of extracranial disease, instead of their presence, was an independent prognostic factor for BCBM patients [22, 30, 31]. Thirdly, the quantity of enrolled patients was relatively small, resulting in the difficulty to achieve baseline matching between groups. Lastly, the lack of external validation limits the generalizability of our findings. In spite of these limitations, our study represents a pioneering effort in analyzing the impact of overall aspects of ECM on the prognosis of BCBM patients, considering a large proportion of clinical trials excluded the patients with brain metastasis.

In summary, many characteristics of the ECM displayed remarkable influence on the prognosis of patients with BCBM. More extracranial sites, both bone and visceral intrusion and progressive lesions indicated dismal survival outcomes. When expanded the presence of extracranial disease to their exact number and control status, the GPA model became more accurate. Other pathological and clinical characteristics were also regarded as independent predictions for long-term survival after BCBM but interfered with some inherent features and remains a subject of debate. To shed more light on this issue, more large scale prospective clinical researches are supposed to be implemented.

## Conclusions

When diagnosed with BCBM, the limited number and stable status of ECM organs portends better life expectancy. These clinical features could be utilized to expand and optimize the Breast-GPA model.

## Electronic supplementary material

Below is the link to the electronic supplementary material.

Supplementary Material 1

## Data Availability

The data presented in this study are available on request from the corresponding author.

## References

[1] Valiente M, Ahluwalia MS, Boire A, Brastianos PK, Goldberg SB, Lee EQ, Le Rhun E, Preusser M, Winkler F, Soffietti R. The Evolving Landscape of Brain Metastasis. Trends Cancer. 2018;4(3):176–96. 29506669 10.1016/j.trecan.2018.01.003 PMC6602095

[2] Kim M, Kizilbash SH, Laramy JK, Gampa G, Parrish KE, Sarkaria JN, Elmquist WF. Barriers to Effective Drug Treatment for Brain metastases: a multifactorial problem in the delivery of Precision Medicine. Pharm Res. 2018;35(9):177. 30003344 10.1007/s11095-018-2455-9 PMC6700736

[3] Boire A, Brastianos PK, Garzia L, Valiente M. Brain metastasis. Nat Rev Cancer. 2020;20(1):4–11. 31780784 10.1038/s41568-019-0220-y

[4] Watase C, Shiino S, Shimoi T, Noguchi E, Kaneda T, Yamamoto Y, Yonemori K, Takayama S, Suto A. Breast Cancer brain metastasis-overview of Disease State, Treatment options and Future perspectives. Cancers (Basel) 2021, 13(5). 10.3390/cancers13051078 PMC7959316 33802424

[5] William J, Gradishar MSM, Jame Abraham R, Aft. Breast Cancer NCCN Clinical Practice Guidelines in Oncology (NCCN Guidelines). 2024, Version 1.2024.

[6] Shen B, Li J, Yang M, Liu K, Zhang J, Li W, Zhang Y, Wang K. Interactive effects of molecular subtypes with tumor size and extracranial metastatic pattern on risk of brain metastasis in breast cancer patients: a population-based study. Cancer Med. 2023;12(6):6547–57. 36353772 10.1002/cam4.5425 PMC10067112

[7] Wu Q, Sun MS, Liu YH, Ye JM, Xu L. Development and external validation of a prediction model for brain metastases in patients with metastatic breast cancer. J Cancer Res Clin Oncol. 2023;149(13):12333–53. 37432458 10.1007/s00432-023-05125-y PMC11797857

[8] Sperduto PW, Kased N, Roberge D, Xu Z, Shanley R, Luo X, Sneed PK, Chao ST, Weil RJ, Suh J, et al. Effect of tumor subtype on survival and the graded prognostic assessment for patients with breast cancer and brain metastases. Int J Radiat Oncol Biol Phys. 2012;82(5):2111–7. 21497451 10.1016/j.ijrobp.2011.02.027 PMC3172400

[9] Barnholtz-Sloan JS, Yu C, Sloan AE, Vengoechea J, Wang M, Dignam JJ, Vogelbaum MA, Sperduto PW, Mehta MP, Machtay M, et al. A nomogram for individualized estimation of survival among patients with brain metastasis. Neuro Oncol. 2012;14(7):910–8. 22544733 10.1093/neuonc/nos087 PMC3379797

[10] Sperduto PW, Mesko S, Li J, Cagney D, Aizer A, Lin NU, Nesbit E, Kruser TJ, Chan J, Braunstein S, et al. Beyond an updated graded Prognostic Assessment (breast GPA): a Prognostic Index and trends in treatment and survival in breast Cancer Brain metastases from 1985 to today. Int J Radiat Oncol Biol Phys. 2020;107(2):334–43. 32084525 10.1016/j.ijrobp.2020.01.051 PMC7276246

[11] Liu Q, Kong X, Wang Z, Wang X, Zhang W, Ai B, Gao R, Fang Y, Wang J. NCCBM, a Nomogram Prognostic Model in breast Cancer patients with brain metastasis. Front Oncol. 2021;11:642677. 33996557 10.3389/fonc.2021.642677 PMC8116746

[12] Leone JP, Lee AV, Brufsky AM. Prognostic factors and survival of patients with brain metastasis from breast cancer who underwent craniotomy. Cancer Med. 2015;4(7):989–94. 25756607 10.1002/cam4.439 PMC4529337

[13] Michel A, Darkwah Oppong M, Rauschenbach L, Dinger TF, Barthel L, Pierscianek D, Wrede KH, Hense J, Pöttgen C, Junker A et al. Prediction of short and long survival after surgery for breast Cancer brain metastases. Cancers (Basel) 2022, 14(6). 10.3390/cancers14061437 PMC8946189 35326590

[14] Sperduto PW, Kased N, Roberge D, Xu Z, Shanley R, Luo X, Sneed PK, Chao ST, Weil RJ, Suh J, et al. Summary report on the graded prognostic assessment: an accurate and facile diagnosis-specific tool to estimate survival for patients with brain metastases. J Clin Oncol. 2012;30(4):419–25. 22203767 10.1200/JCO.2011.38.0527 PMC3269967

[15] Subbiah IM, Lei X, Weinberg JS, Sulman EP, Chavez-MacGregor M, Tripathy D, Gupta R, Varma A, Chouhan J, Guevarra RP, et al. Validation and development of a modified breast graded Prognostic Assessment as a Tool for Survival in patients with breast Cancer and brain metastases. J Clin Oncol. 2015;33(20):2239–45. 25987700 10.1200/JCO.2014.58.8517 PMC5098846

[16] Sperduto PW, Mesko S, Li J, Cagney D, Aizer A, Lin NU, Nesbit E, Kruser TJ, Chan J, Braunstein S, et al. Survival in patients with brain metastases: Summary Report on the updated diagnosis-specific graded Prognostic Assessment and Definition of the eligibility quotient. J Clin Oncol. 2020;38(32):3773–84. 32931399 10.1200/JCO.20.01255 PMC7655019

[17] Xiong Y, Cao H, Zhang Y, Pan Z, Dong S, Wang G, Wang F, Li X. Nomogram-predicted survival of breast Cancer brain metastasis: a SEER-Based Population Study. World Neurosurg. 2019;128:e823–34. 31096027 10.1016/j.wneu.2019.04.262

[18] Nie Y, Ying B, Lu Z, Sun T, Sun G. Predicting survival and prognosis of postoperative breast cancer brain metastasis: a population-based retrospective analysis. Chin Med J (Engl). 2023;136(14):1699–707. 37257115 10.1097/CM9.0000000000002674 PMC10344505

[19] Yang Y, Zhang L, Tian W, Li Y, Qin Q, Mao Y, Liu X, Hong J, Hu L, Zeng Q, et al. Prognosis prediction and risk factors for triple-negative breast cancer patients with brain metastasis: a population-based study. Cancer Med. 2023;12(7):7951–61. 36629093 10.1002/cam4.5575 PMC10134296

[20] Kim YJ, Kim JS, Kim IA. Molecular subtype predicts incidence and prognosis of brain metastasis from breast cancer in SEER database. J Cancer Res Clin Oncol. 2018;144(9):1803–16. 29971531 10.1007/s00432-018-2697-2 PMC11813522

[21] Noteware L, Broadwater G, Dalal N, Alder L, Herndon Ii JE, Floyd S, Giles W, Van Swearingen AED, Anders CK, Sammons S. Brain metastasis as the first and only metastatic relapse site portends worse survival in patients with advanced HER2 + breast cancer. Breast Cancer Res Treat. 2023;197(2):425–34. 36403183 10.1007/s10549-022-06799-7

[22] Matsuo S, Watanabe J, Mitsuya K, Hayashi N, Nakasu Y, Hayashi M. Brain metastasis in patients with metastatic breast cancer in the real world: a single-institution, retrospective review of 12-year follow-up. Breast Cancer Res Treat. 2017;162(1):169–79. 28084583 10.1007/s10549-017-4107-x

[23] Yap YS, Cornelio GH, Devi BC, Khorprasert C, Kim SB, Kim TY, Lee SC, Park YH, Sohn JH, Sutandyo N, et al. Brain metastases in Asian HER2-positive breast cancer patients: anti-HER2 treatments and their impact on survival. Br J Cancer. 2012;107(7):1075–82. 22918394 10.1038/bjc.2012.346 PMC3461152

[24] Niikura N, Hayashi N, Masuda N, Takashima S, Nakamura R, Watanabe K, Kanbayashi C, Ishida M, Hozumi Y, Tsuneizumi M, et al. Treatment outcomes and prognostic factors for patients with brain metastases from breast cancer of each subtype: a multicenter retrospective analysis. Breast Cancer Res Treat. 2014;147(1):103–12. 25106661 10.1007/s10549-014-3090-8

[25] Leone JP, Leone J, Zwenger AO, Iturbe J, Leone BA, Vallejo CT. Prognostic factors and survival according to tumour subtype in women presenting with breast cancer brain metastases at initial diagnosis. Eur J Cancer. 2017;74:17–25. 28335884 10.1016/j.ejca.2016.12.015

[26] Curigliano G, Mueller V, Borges V, Hamilton E, Hurvitz S, Loi S, Murthy R, Okines A, Paplomata E, Cameron D, et al. Tucatinib versus placebo added to trastuzumab and capecitabine for patients with pretreated HER2 + metastatic breast cancer with and without brain metastases (HER2CLIMB): final overall survival analysis. Annals Oncology: Official J Eur Soc Med Oncol. 2022;33(3):321–9. 10.1016/j.annonc.2021.12.005 34954044

[27] Harbeck N, Huang CS, Hurvitz S, Yeh DC, Shao Z, Im SA, Jung KH, Shen K, Ro J, Jassem J, et al. Afatinib plus Vinorelbine versus trastuzumab plus vinorelbine in patients with HER2-overexpressing metastatic breast cancer who had progressed on one previous trastuzumab treatment (LUX-Breast 1): an open-label, randomised, phase 3 trial. Lancet Oncol. 2016;17(3):357–66. 26822398 10.1016/S1470-2045(15)00540-9

[28] Cortés J, Hurvitz SA, Im SA, Iwata H, Curigliano G, Kim SB, Chiu JWY, Pedrini JL, Li W, Yonemori K et al. Trastuzumab deruxtecan versus trastuzumab emtansine in HER2-positive metastatic breast cancer: long-term survival analysis of the DESTINY-Breast03 trial. Nat Med 2024. 10.1038/s41591-024-03021-7 PMC11333275 38825627

[29] Krop IE, Kim SB, González-Martín A, LoRusso PM, Ferrero JM, Smitt M, Yu R, Leung AC, Wildiers H. Trastuzumab emtansine versus treatment of physician’s choice for pretreated HER2-positive advanced breast cancer (TH3RESA): a randomised, open-label, phase 3 trial. Lancet Oncol. 2014;15(7):689–99. 24793816 10.1016/S1470-2045(14)70178-0

[30] Aoyagi K, Higuchi Y, Matsunaga S, Serizawa T, Yomo S, Aiyama H, Nagano O, Kondoh T, Kenai H, Shuto T, et al. Impact of breast cancer subtype on clinical outcomes after Gamma Knife radiosurgery for brain metastases from breast cancer: a multi-institutional retrospective study (JLGK1702). Breast Cancer Res Treat. 2020;184(1):149–59. 32737714 10.1007/s10549-020-05835-8

[31] van Schie P, Rijksen BLT, Bot M, Wiersma T, Merckel LG, Brandsma D, Compter A, de Witt Hamer PC, Post R, Borst GR. Optimizing treatment of brain metastases in an era of novel systemic treatments: a single center consecutive series. J Neurooncol 2023. 10.1007/s11060-023-04343-1 PMC10322956 37266846

