# Supplementary material for: Development of graded prognostic assessment for breast Cancer brain metastasis incorporating extracranial metastatic features: a retrospective analysis of 284 patients
Source: BMC Cancer. 2024 Oct 10;24:1262. doi: 10.1186/s12885-024-12983-3 (PMC11465582; doi:10.1186/s12885-024-12983-3)
Supplement: Supplementary file 1 — Supplementary Material 1 [file 12885_2024_12983_MOESM1_ESM.docx]

**Supplementary Materials**

**Table S1.** Baseline characteristics of patients stratified by the presence of extracranial metastasis.

**Table S2.** Baseline characteristics of patients stratified by the number of extracranial metastatic organs.

**Table S3.** Baseline characteristics of patients stratified by the control status of extracranial metastasis.

**Table S4.** Comparison of 24-Month RMST Between Original and New Breast-GPA Models Across Score Groups.

| **Characteristics** | With Extracranial Metastases  (n=236) | | Without Extracranial Metastases  (n=48) | | p-value |
| --- | --- | --- | --- | --- | --- |
|  | No. | Percent (%) | No. | Percent (%) |  |
| **Age, years** |  |  |  |  | 0.562 |
| ＜40 | 61 | 0.26 | 15 | 0.31 |  |
| 40-59 | 152 | 0.64 | 27 | 0.56 |  |
| 60-79 | 23 | 0.10 | 6 | 0.13 |  |
| **Menopausal Status** |  |  |  |  | 0.770 |
| Premenopausal | 129 | 0.55 | 27 | 0.57 |  |
| Postmenopausal | 105 | 0.45 | 20 | 0.43 |  |
| **Performance Status** |  |  |  |  | 0.582 |
| KPS 80-100 / ECOG 0-1 | 142 | 0.60 | 25 | 0.52 |  |
| KPS ≤70 / ECOG 2-3 | 36 | 0.15 | 9 | 0.19 |  |
| Unknown | 58 | 0.25 | 14 | 0.29 |  |
| **Grade** |  |  |  |  | 0.394 |
| Grade Ⅰ-Ⅱ | 100 | 0.42 | 19 | 0.40 |  |
| Grade Ⅲ | 60 | 0.25 | 9 | 0.19 |  |
| Unknown | 76 | 0.32 | 20 | 0.42 |  |
| **T Stage** |  |  |  |  | 0.355 |
| Tis/T0-2 | 144 | 0.61 | 28 | 0.58 |  |
| T3-T4 | 33 | 0.14 | 4 | 0.08 |  |
| Unknown | 59 | 0.25 | 16 | 0.33 |  |
| **N Stage** |  |  |  |  | 0.373 |
| N0-N1 | 107 | 0.45 | 19 | 0.40 |  |
| N2-N3 | 90 | 0.38 | 17 | 0.35 |  |
| Unknown | 39 | 0.17 | 12 | 0.25 |  |
| **M Stage** |  |  |  |  | **0.040** |
| M0 | 197 | 0.84 | 44 | 0.92 |  |
| M1 | 37 | 0.16 | 2 | 0.04 |  |
| **TNM Stage** |  |  |  |  | 0.456 |
| Stage 0-Ⅱ | 83 | 0.35 | 16 | 0.33 |  |
| Stage Ⅲ-Ⅳ | 112 | 0.47 | 20 | 0.42 |  |
| Unknown | 41 | 0.17 | 12 | 0.25 |  |
| **HR Status** |  |  |  |  | 0.530 |
| Positive | 144 | 0.62 | 27 | 0.57 |  |
| Negative | 87 | 0.38 | 20 | 0.43 |  |
| **HER2 Status** |  |  |  |  | 0.870 |
| Positive | 113 | 0.50 | 22 | 0.49 |  |
| Negative | 112 | 0.50 | 23 | 0.51 |  |
| **Neoadjuvant Therapy** |  |  |  |  | 0.728 |
| Yes | 49 | 0.21 | 11 | 0.23 |  |
| No | 183 | 0.79 | 36 | 0.77 |  |
| **Surgery** |  |  |  |  | **0.044** |
| Yes-Radical Mastectomy | 164 | 0.71 | 26 | 0.58 |  |
| Yes-Conserving Surgery* | 44 | 0.19 | 16 | 0.36 |  |
| No | 24 | 0.10 | 3 | 0.07 |  |

| **Characteristics** | With Extracranial Metastases  (n=236) | | Without Extracranial Metastases  (n=48) | | p-value |
| --- | --- | --- | --- | --- | --- |
|  | No. | Percent (%) | No. | Percent (%) |  |
| **Adjuvant Chemotherapy** |  |  |  |  | 0.565 |
| Yes | 174 | 0.77 | 32 | 0.74 |  |
| No | 30 | 0.13 | 8 | 0.19 |  |
| Without Surgery | 23 | 0.10 | 3 | 0.07 |  |
| **Adjuvant Radiotherapy** |  |  |  |  | 0.423 |
| Yes | 116 | 0.50 | 28 | 0.60 |  |
| No | 91 | 0.39 | 16 | 0.34 |  |
| Without Surgery | 25 | 0.11 | 3 | 0.06 |  |
| **DFS after Surgery** |  |  |  |  | 0.933 |
| ≤60m | 167 | 0.71 | 36 | 0.75 |  |
| ＞60m | 37 | 0.16 | 7 | 0.15 |  |
| Without Surgery | 19 | 0.08 | 3 | 0.06 |  |
| Unknown | 13 | 0.06 | 2 | 0.04 |  |
| **Diagnostic Mode** |  |  |  |  | **＜0.001** |
| Symptomatic diagnosis | 100 | 0.42 | 34 | 0.71 |  |
| Occasional in imaging | 136 | 0.58 | 14 | 0.29 |  |
| **Number of BM** |  |  |  |  | **0.003** |
| 1 | 70 | 0.31 | 25 | 0.54 |  |
| ≥2 | 153 | 0.69 | 21 | 0.46 |  |
| **Maximum of BM Diameter** |  |  |  |  | **0.003** |
| ＜3cm* | 123 | 0.52 | 14 | 0.29 |  |
| ≥3cm* | 37 | 0.16 | 16 | 0.33 |  |
| Unknown | 76 | 0.32 | 18 | 0.38 |  |
| **Radiotherapy for BM** |  |  |  |  | 0.099 |
| SRS alone | 55 | 0.25 | 16 | 0.36 |  |
| WBRT alone | 95 | 0.42 | 10 | 0.23 |  |
| SRS plus WBRT | 19 | 0.08 | 5 | 0.11 |  |
| No Radiotherapy | 55 | 0.25 | 13 | 0.30 |  |
| **Surgical Intervention for BM** |  |  |  |  | **0.003** |
| Yes | 26 | 0.11 | 13 | 0.27 |  |
| No | 209 | 0.89 | 35 | 0.73 |  |
| **Chemotherapy after BM** |  |  |  |  | 0.670 |
| Yes | 189 | 0.82 | 39 | 0.85 |  |
| No | 41 | 0.18 | 7 | 0.15 |  |
| **Endocrine Therapy after BM*** |  |  |  |  | 0.813 |
| ET for HR+ tumors | 47 | 0.21 | 8 | 0.18 |  |
| no ET for HR+ tumors | 95 | 0.42 | 18 | 0.40 |  |
| HR- tumors | 85 | 0.37 | 19 | 0.42 |  |
| **Targeted Therapy after BM*** |  |  |  |  | 0.095 |
| TT for HER2+ tumors | 88 | 0.40 | 12 | 0.29 |  |
| no TT for HER2+ tumors | 23 | 0.10 | 9 | 0.21 |  |
| HER2- tumors | 110 | 0.50 | 21 | 0.50 |  |

**Supplementary Table 1.** The baseline characteristics of patients stratified by the presence of extracranial metastasis (p-values lower than 0.05 were bold). Abbreviations: KPS, Karnofsky performance status; ECOG, Eastern Cooperative Oncology Group; HR, hormone receptor; HER2, human epidermal growth factor receptor 2; DFS, disease-free survival; BM, brain metastasis; SRS, stereotactic radiosurgery; WBRT, whole-brain radiotherapy; ET, endocrine therapy; TT, targeted therapy. *The subgroups mainly contributing to the significant difference in the analysis involving more than two groups.

| **Characteristics** | Without Extracranial  Metastases (n=48) | | With Extracranial  Metastases (1-3) (n=189) | | With Extracranial  Metastases (≥4) (n=47) | | p-value |
| --- | --- | --- | --- | --- | --- | --- | --- |
|  | No. | Percent (%) | No. | Percent (%) | No. | Percent (%) |  |
| **Age, years** |  |  |  |  |  |  | 0.782 |
| ＜40 | 15 | 0.31 | 49 | 0.26 | 12 | 0.26 |  |
| 40-59 | 27 | 0.56 | 123 | 0.65 | 29 | 0.62 |  |
| 60-79 | 6 | 0.13 | 17 | 0.09 | 6 | 0.13 |  |
| **Menopausal Status** |  |  |  |  |  |  | 0.924 |
| Premenopausal | 27 | 0.57 | 105 | 0.56 | 24 | 0.53 |  |
| Postmenopausal | 20 | 0.43 | 84 | 0.44 | 21 | 0.47 |  |
| **Performance Status** |  |  |  |  |  |  | 0.600 |
| KPS 80-100 / ECOG 0-1 | 25 | 0.52 | 115 | 0.61 | 27 | 0.57 |  |
| KPS ≤70 / ECOG 2-3 | 9 | 0.19 | 26 | 0.14 | 10 | 0.21 |  |
| Unknown | 14 | 0.29 | 48 | 0.25 | 10 | 0.21 |  |
| **Grade** |  |  |  |  |  |  | 0.660 |
| Grade Ⅰ-Ⅱ | 19 | 0.40 | 79 | 0.42 | 21 | 0.45 |  |
| Grade Ⅲ | 9 | 0.19 | 47 | 0.25 | 13 | 0.28 |  |
| Unknown | 20 | 0.42 | 63 | 0.33 | 13 | 0.28 |  |
| **T Stage** |  |  |  |  |  |  | **0.044** |
| Tis/T0-2* | 28 | 0.58 | 107 | 0.57 | 37 | 0.79 |  |
| T3-T4 | 4 | 0.08 | 29 | 0.15 | 4 | 0.09 |  |
| Unknown | 16 | 0.33 | 53 | 0.28 | 6 | 0.13 |  |
| **N Stage** |  |  |  |  |  |  | **0.047** |
| N0-N1 | 19 | 0.40 | 79 | 0.42 | 28 | 0.60 |  |
| N2-N3 | 17 | 0.35 | 73 | 0.39 | 17 | 0.36 |  |
| Unknown* | 12 | 0.25 | 37 | 0.20 | 2 | 0.04 |  |
| **M Stage** |  |  |  |  |  |  | 0.097 |
| M0 | 44 | 0.92 | 156 | 0.83 | 41 | 0.87 |  |
| M1 | 2 | 0.04 | 31 | 0.17 | 6 | 0.13 |  |
| **TNM Stage** |  |  |  |  |  |  | **0.006** |
| Stage 0-Ⅱ* | 16 | 0.33 | 57 | 0.30 | 26 | 0.55 |  |
| Stage Ⅲ-Ⅳ | 20 | 0.42 | 93 | 0.49 | 19 | 0.40 |  |
| Unknown* | 12 | 0.25 | 39 | 0.21 | 2 | 0.04 |  |
| **HR Status** |  |  |  |  |  |  | 0.236 |
| Positive | 27 | 0.57 | 110 | 0.60 | 34 | 0.72 |  |
| Negative | 20 | 0.43 | 74 | 0.40 | 13 | 0.28 |  |
| **HER2 Status** |  |  |  |  |  |  | **0.044** |
| Positive | 22 | 0.49 | 97 | 0.54 | 16 | 0.34 |  |
| Negative | 23 | 0.51 | 81 | 0.46 | 31 | 0.66 |  |
| **Neoadjuvant Therapy** |  |  |  |  |  |  | 0.311 |
| Yes | 11 | 0.23 | 43 | 0.23 | 6 | 0.13 |  |
| No | 36 | 0.77 | 143 | 0.77 | 40 | 0.87 |  |
| **Surgery** |  |  |  |  |  |  | 0.162 |
| Yes-Radical Mastectomy | 26 | 0.58 | 130 | 0.70 | 34 | 0.74 |  |
| Yes-Conserving Surgery | 16 | 0.36 | 36 | 0.19 | 8 | 0.17 |  |
| No | 3 | 0.07 | 20 | 0.11 | 4 | 0.09 |  |
|  |  |  |  |  |  |  |  |
| **Characteristics** | Without Extracranial  Metastases (n=48) | | With Extracranial  Metastases (1-3) (n=189) | | Without Extracranial  Metastases (≥4) (n=47) | | p-value |
|  | No. | Percent (%) | No. | Percent (%) | No. | Percent (%) |  |
| **Adjuvant Chemotherapy** |  |  |  |  |  |  | 0.283 |
| Yes | 32 | 0.74 | 135 | 0.74 | 39 | 0.87 |  |
| No | 8 | 0.19 | 28 | 0.15 | 2 | 0.04 |  |
| Without Surgery | 3 | 0.07 | 19 | 0.10 | 4 | 0.09 |  |
| **Adjuvant Radiotherapy** |  |  |  |  |  |  | 0.766 |
| Yes | 28 | 0.60 | 94 | 0.51 | 22 | 0.48 |  |
| No | 16 | 0.34 | 72 | 0.39 | 19 | 0.41 |  |
| Without Surgery | 3 | 0.06 | 20 | 0.11 | 5 | 0.11 |  |
| **DFS after Surgery** |  |  |  |  |  |  | 0.936 |
| ≤60m | 36 | 0.75 | 133 | 0.70 | 34 | 0.72 |  |
| ＞60m | 7 | 0.15 | 29 | 0.15 | 8 | 0.17 |  |
| Without Surgery | 3 | 0.06 | 15 | 0.08 | 4 | 0.09 |  |
| Unknown | 2 | 0.04 | 12 | 0.06 | 1 | 0.02 |  |
| **Diagnostic Mode** |  |  |  |  |  |  | **0.002** |
| Symptomatic diagnosis | 34 | 0.71 | 80 | 0.42 | 20 | 0.43 |  |
| Occasional in imaging | 14 | 0.29 | 109 | 0.58 | 27 | 0.57 |  |
| **Number of BM** |  |  |  |  |  |  | **0.041** |
| 1 | 25 | 0.54 | 61 | 0.34 | 16 | 0.36 |  |
| ≥2 | 21 | 0.46 | 117 | 0.66 | 29 | 0.64 |  |
| **Maximum of BM Diameter** |  |  |  |  |  |  | **0.014** |
| ＜3cm* | 14 | 0.29 | 96 | 0.51 | 27 | 0.57 |  |
| ≥3cm* | 16 | 0.33 | 32 | 0.17 | 5 | 0.11 |  |
| Unknown | 18 | 0.38 | 61 | 0.32 | 15 | 0.32 |  |
| **Radiotherapy for BM** |  |  |  |  |  |  | 0.143 |
| SRS alone | 16 | 0.36 | 48 | 0.27 | 7 | 0.15 |  |
| WBRT alone* | 10 | 0.23 | 71 | 0.40 | 24 | 0.52 |  |
| SRS plus WBRT | 5 | 0.11 | 15 | 0.08 | 4 | 0.09 |  |
| No Radiotherapy | 13 | 0.30 | 44 | 0.25 | 11 | 0.24 |  |
| **Surgical Intervention for BM** |  |  |  |  |  |  | **0.004** |
| Yes | 13 | 0.27 | 24 | 0.13 | 2 | 0.04 |  |
| No | 35 | 0.73 | 164 | 0.87 | 45 | 0.96 |  |
| **Chemotherapy after BM** |  |  |  |  |  |  | 0.765 |
| Yes | 39 | 0.85 | 149 | 0.81 | 40 | 0.85 |  |
| No | 7 | 0.15 | 34 | 0.19 | 7 | 0.15 |  |
| **Endocrine Therapy after BM*** |  |  |  |  |  |  | 0.488 |
| ET for HR+ tumors | 8 | 0.18 | 34 | 0.19 | 13 | 0.28 |  |
| no ET for HR+ tumors | 18 | 0.40 | 74 | 0.41 | 21 | 0.45 |  |
| HR- tumors | 19 | 0.42 | 72 | 0.40 | 13 | 0.28 |  |
| **Targeted Therapy after BM*** |  |  |  |  |  |  | **0.024** |
| TT for HER2+ tumors | 12 | 0.29 | 76 | 0.44 | 12 | 0.26 |  |
| no TT for HER2+ tumors | 9 | 0.21 | 19 | 0.11 | 4 | 0.09 |  |
| HER2- tumors* | 21 | 0.50 | 79 | 0.45 | 31 | 0.66 |  |

**Supplementary Table 2.** Baseline characteristics of patients stratified by the number of extracranial metastatic organs (p-values lower than 0.05 were bold). Abbreviations: KPS, Karnofsky performance status; ECOG, Eastern Cooperative Oncology Group; HR, hormone receptor; HER2, human epidermal growth factor receptor 2; DFS, disease-free survival; BM, brain metastasis; SRS, stereotactic radiosurgery; WBRT, whole-brain radiotherapy; ET, endocrine therapy; TT, targeted therapy.*The subgroups mainly contributing to the significant difference in the analysis involving more than two groups.

| **Characteristics** | With Controlled Extracranial Metastases (n=160) | | With Uncontrolled Extracranial Metastases (n=120) | | p-value |
| --- | --- | --- | --- | --- | --- |
|  | No. | Percent (%) | No. | Percent (%) |  |
| **Age, years** |  |  |  |  | 0.106 |
| ＜40 | 50 | 0.31 | 25 | 0.21 |  |
| 40-59 | 97 | 0.61 | 80 | 0.67 |  |
| 60-79 | 13 | 0.08 | 15 | 0.13 |  |
| **Menopausal Status** |  |  |  |  | 0.929 |
| Premenopausal | 89 | 0.56 | 65 | 0.54 |  |
| Postmenopausal | 71 | 0.44 | 53 | 0.44 |  |
| **Performance Status** |  |  |  |  | 0.761 |
| KPS 80-100 / ECOG 0-1 | 96 | 0.60 | 67 | 0.56 |  |
| KPS ≤70 / ECOG 2-3 | 24 | 0.15 | 21 | 0.18 |  |
| Unknown | 40 | 0.25 | 32 | 0.27 |  |
| **Grade** |  |  |  |  | 0.802 |
| Grade Ⅰ-Ⅱ | 67 | 0.42 | 51 | 0.43 |  |
| Grade Ⅲ | 37 | 0.23 | 31 | 0.26 |  |
| Unknown | 56 | 0.35 | 38 | 0.32 |  |
| **T Stage** |  |  |  |  | 0.349 |
| Tis/T0-2 | 102 | 0.64 | 68 | 0.57 |  |
| T3-T4 | 17 | 0.11 | 19 | 0.16 |  |
| Unknown | 41 | 0.26 | 33 | 0.28 |  |
| **N Stage** |  |  |  |  | 0.655 |
| N0-N1 | 75 | 0.47 | 50 | 0.42 |  |
| N2-N3 | 59 | 0.37 | 47 | 0.39 |  |
| Unknown | 26 | 0.16 | 23 | 0.19 |  |
| **M Stage** |  |  |  |  | 0.440 |
| M0 | 140 | 0.88 | 101 | 0.84 |  |
| M1 | 19 | 0.12 | 18 | 0.15 |  |
| **TNM Stage** |  |  |  |  | 0.785 |
| Stage 0-Ⅱ | 59 | 0.37 | 40 | 0.33 |  |
| Stage Ⅲ-Ⅳ | 71 | 0.44 | 58 | 0.48 |  |
| Unknown | 30 | 0.19 | 22 | 0.18 |  |
| **HR Status** |  |  |  |  | 0.989 |
| Positive | 97 | 0.61 | 73 | 0.61 |  |
| Negative | 60 | 0.38 | 45 | 0.38 |  |
| **HER2 Status** |  |  |  |  | 0.129 |
| Positive | 82 | 0.51 | 53 | 0.44 |  |
| Negative | 68 | 0.43 | 64 | 0.53 |  |
| **Neoadjuvant Therapy** |  |  |  |  | 0.940 |
| Yes | 34 | 0.21 | 25 | 0.21 |  |
| No | 125 | 0.78 | 94 | 0.78 |  |
| **Surgery** |  |  |  |  | **0.041** |
| Yes-Radical Mastectomy | 108 | 0.68 | 82 | 0.68 |  |
| Yes-Conserving Surgery | 39 | 0.24 | 20 | 0.17 |  |
| No* | 10 | 0.06 | 17 | 0.14 |  |
| **Adjuvant Chemotherapy** |  |  |  |  | 0.128 |
| Yes | 120 | 0.75 | 87 | 0.73 |  |
| No | 23 | 0.14 | 14 | 0.12 |  |
| Without Surgery* | 10 | 0.06 | 16 | 0.13 |  |
|  |  |  |  |  |  |
| **Characteristics** | With Controlled Extracranial Metastases (n=160) | | With Uncontrolled Extracranial Metastases (n=120) | | p-value |
|  | No. | Percent (%) | No. | Percent (%) |  |
| **Adjuvant Radiotherapy** |  |  |  |  | 0.110 |
| Yes | 83 | 0.52 | 61 | 0.51 |  |
| No | 65 | 0.41 | 41 | 0.34 |  |
| Without Surgery | 11 | 0.07 | 17 | 0.14 |  |
| **DFS after Surgery** |  |  |  |  | **0.001** |
| ≤60m | 115 | 0.72 | 88 | 0.73 |  |
| ＞60m* | 32 | 0.20 | 12 | 0.10 |  |
| Without Surgery* | 5 | 0.03 | 17 | 0.14 |  |
| Unknown | 8 | 0.05 | 3 | 0.03 |  |
| **Diagnostic Mode** |  |  |  |  | **＜0.001** |
| Symptomatic diagnosis | 91 | 0.57 | 40 | 0.33 |  |
| Occasional in imaging | 69 | 0.43 | 80 | 0.67 |  |
| **Number of BM** |  |  |  |  | 0.306 |
| 1 | 74 | 0.46 | 49 | 0.41 |  |
| ≥2 | 76 | 0.48 | 65 | 0.54 |  |
| **Maximum of BM Diameter** |  |  |  |  | 0.055 |
| ＜3cm | 70 | 0.44 | 63 | 0.53 |  |
| ≥3cm | 38 | 0.24 | 15 | 0.13 |  |
| Unknown | 52 | 0.33 | 42 | 0.35 |  |
| **Radiotherapy for BM** |  |  |  |  | 0.301 |
| SRS alone | 36 | 0.23 | 35 | 0.29 |  |
| WBRT alone | 54 | 0.34 | 49 | 0.41 |  |
| SRS plus WBRT | 16 | 0.10 | 8 | 0.07 |  |
| No Radiotherapy | 42 | 0.26 | 25 | 0.21 |  |
| **Surgical Intervention for BM** |  |  |  |  | **＜0.001** |
| Yes | 34 | 0.21 | 6 | 0.05 |  |
| No | 126 | 0.79 | 114 | 0.95 |  |
| **Chemotherapy after BM** |  |  |  |  | **0.049** |
| Yes | 121 | 0.76 | 103 | 0.86 |  |
| No | 34 | 0.21 | 15 | 0.13 |  |
| **Endocrine Therapy after BM*** |  |  |  |  | 0.970 |
| ET for HR+ tumors | 30 | 0.19 | 24 | 0.20 |  |
| no ET for HR+ tumors | 65 | 0.41 | 48 | 0.40 |  |
| HR- tumors | 59 | 0.37 | 44 | 0.37 |  |
| **Targeted Therapy after BM*** |  |  |  |  | 0.079 |
| TT for HER2+ tumors | 59 | 0.37 | 40 | 0.33 |  |
| no TT for HER2+ tumors | 21 | 0.13 | 12 | 0.10 |  |
| HER2- tumors | 56 | 0.35 | 64 | 0.53 |  |

**Supplementary Table 3.** Baseline characteristics of patients stratified by the extracranial metastasis status (p-values lower than 0.05 were bold). Abbreviations: KPS, Karnofsky performance status; ECOG, Eastern Cooperative Oncology Group; HR, hormone receptor; HER2, human epidermal growth factor receptor 2; DFS, disease-free survival; BM, brain metastasis; SRS, stereotactic radiosurgery; WBRT, whole-brain radiotherapy; ET, endocrine therapy; TT, targeted therapy.*The subgroups mainly contributing to the significant difference in the analysis involving more than two groups.

| Model | Score Group | 24-month RMST | SE | 95% CI Lower Limit | 95% CI Upper Limit |
| --- | --- | --- | --- | --- | --- |
| Original | 0-1.0 | 14.668 | 1.802 | 11.137 | 18.199 |
|  | 1.5-2.0 | 16.760 | 0.902 | 14.991 | 18.528 |
|  | 2.5-3.0 | 19.519 | 0.723 | 18.101 | 20.937 |
|  | 3.5-4.0 | 17.923 | 1.834 | 14.329 | 21.517 |
| New | 0-1.0 | 12.762 | 2.316 | 8.223 | 17.301 |
|  | 1.5-2.0 | 15.868 | 0.997 | 13.913 | 17.823 |
|  | 2.5-3.0 | 18.914 | 0.745 | 17.454 | 20.375 |
|  | 3.5-4.0 | 20.439 | 1.083 | 18.317 | 22.561 |

**Supplementary Table 4.** Comparison of 24-Month RMST Between Original and New Breast-GPA Models Across Score Groups
